# Patient satisfaction with a teleradiology service in general practice

**DOI:** 10.1186/s12875-016-0418-y

**Published:** 2016-02-10

**Authors:** Jac J. W. M. Jacobs, Rianne Ekkelboom, Jan P. A. M. Jacobs, Thys van der Molen, Robbert Sanderman

**Affiliations:** General Practice, 9162 ER Ballum, Ameland, The Netherlands; Health Psychology Section, University Medical Centre Groningen, University of Groningen, 9712 CP Groningen, The Netherlands; Faculty of Economics and Business, University of Groningen, 9700 AV Groningen, The Netherlands; Department of Primary Care, University Medical Centre Groningen, University of Groningen, 9712 CP Groningen, The Netherlands; Department of Psychology, Health and Technology, University of Twente, 7522 NB Enschede, The Netherlands

**Keywords:** Teleradiology, Patient satisfaction, General practice, Family practice

## Abstract

**Background:**

Accessibility to secondary health services is not always easy for patients who live at a great distance of hospital. In these circumstances, transferring diagnostic tools and treatment options to primary care could prove beneficial for patients. To do so, the quality of medical care and the costs and benefits of the approach need to be assessed. However, the patient perspective is equally important, offering important insights.

**Methods:**

In a cross-sectional study we investigate the satisfaction of patients toward a new teleradiology facility offered a general practice on Ameland, an island in the Netherlands.

A questionnaire was created based on the Dutch version of the Patient Satisfaction Questionnaire III and completed by all patients after receiving an x-ray in primary care between June 1, 2007 and June 1, 2009. Those who received more than one x-ray in that period were included only once. The technical and interpersonal skills of doctors were rated out the sum score of the questionnaire namely 25 and 30, respectively. Analysis of variance (ANOVA) was used to analyze the differences between the means of the satisfaction subscales and the patient characteristics.

**Results:**

The response proportion was after reminder 65 % (381/587 patients). Satisfaction with the technical skills of the doctor providing the teleradiology service was 22.4 ± 3.7, while satisfaction with the interpersonal skills of the doctor during the diagnostic phase was 26.8 ± 3.8. Island residents, the elderly, and those with no history of trauma were more satisfied with the technical and interpersonal aspects of the consultation than non-residents, younger patients, and those with a history of trauma.

**Conclusion:**

Patients in the island community of Ameland experienced high levels of satisfaction with the teleradiology service offered in primary care.

**Electronic supplementary material:**

The online version of this article (doi:10.1186/s12875-016-0418-y) contains supplementary material, which is available to authorized users.

## Background

The debate as to whether traditional secondary care services can be transferred to primary care has received renewed impetus, not only because of the need to control spiraling healthcare costs [1–3] but also because of developments in the available technology [4–8]. Indeed, it is now possible for the general practitioner (GP) and the specialist to discuss and determine treatment options through the exchange of electronic data files. Therefore, patients do not necessarily have to attend hospital for specialist diagnostic assessment. This approach is, already commonplace in cardiology and dermatology in the Netherlands where electrocardiograms and images of skin abnormalities, respectively, are sent digitally from the GP to the specialist for diagnosis and treatment [9, 10]. Another potential area where traditional secondary care services can be introduced to primary care is radiological examination. Teleradiology can be of particular benefit in remote areas.

The present article deals with patient satisfaction of the x-ray and teleradiology service offered in primary care on the Dutch island of Ameland. We showed already that the introduction of teleradiology in general practice has reduced the costs for both the healthcare provider and the patient [11], the number of missed fractures, the unnecessary travel to the hospital with an increase of the treatment in the general practice of normally hospital patients [12]. Given these outcomes it is also very important to investigate whether the patients appreciate such a teleradiology facility. To be particular, the aim of this article is to investigate the satisfaction of patients toward a new teleradiology facility offered in primary care in an island community.

Literature on the use of teleradiology in primary care is scarce [4–8], and to our knowledge only one study has reported the views of patients: a General Practice in Otta, Norway, communicates via teleradiology with the hospital in Lillehammer at a distance of 115 km [7]. Of note, a majority of patients (90 %) preferred an x-ray examination in Otta; only 3 % preferred an examination in Lillehammer. Patient satisfaction surveys have been used far more often in the study of telemedicine [13–16], particularly in the form of teledermatology [17–19] and teleconsultation [20]. Again, these studies report very high levels of patient satisfaction with the service [17–21].

It is known from family practice surveys that the continuity of the doctor-patient relationship affects patient satisfaction, as do various sociodemographic factors such as age, sex, education level, and whether or not the patient is seen by their usual doctor [22–25]. This is where teleradiology may be perceived as most useful by patients, with the GP able to offer diagnostic procedures and treatment without the need for time-consuming referrals to hospital. Assessment should ideally not only include the general levels of satisfaction with the service but also the satisfaction with aspects related to the doctor-patient relationship (the interpersonal aspects of care) and the technical skills of GPs providing the service.

## Method*s*

### Study design and data collection

We conducted a cross-sectional survey to analyze patient satisfaction with the teleradiology service between June 1, 2007 and June 1, 2009. All patients who had an x-ray in primary care during the study period were included. Those who received more than one x-ray in that period were included only once. Patients or their representatives (eg. of children and patients with dementia) were asked to provide informed consent after making the x-ray on the general practice. After informed consent, a few weeks later a questionnaire was sent to their home addresses and was filled in at home by the patient themselves or by their representatives. The completed questionnaires were returned in pre-paid envelopes to an independent researcher. This procedure was used to guarantee patient anonymity and to limit the potential for response bias. A reminder was sent after three months to all patients. Patients who did not agree with the informed consent did not receive a questionnaire and were excluded. The subscales of the questionnaire that were not completely filled in by the patients were also excluded.

### Setting

Ameland is a Dutch island with a population of 3500 inhabitants that increases twenty-fold with an influx of tourists during the peak season. There are only two general practices on the island, with the nearest hospital being located on the mainland, requiring a minimum travel time of four hours. Therefore, a teleradiology service was developed, with the facilities located in one of the general practices, but with the service accessible to all patients regardless of practice or tourist status.

X-rays are taken at the facility by a certified radiographer and digitally transmitted to the mainland hospital in Dokkum where evaluation and interpretation are performed by a radiologist or surgeon. This expert review service is available 24 h a day for emergencies and during daytime working hours for routine imaging. Moreover, the radiologist always responds the same day and, if necessary, direct by phone with additional instructions for the radiographer. The teleradiology service is indicated for trauma (e.g., fractures) and non-trauma (e.g., hip, knee, or lung imaging) in preparation for surgery (e.g., coxarthrosis) or for monitoring pulmonary pathology (e.g., lung carcinoma).

Quality assurance is maintained through continuous feedback about the quality of x-rays by the radiologist, and by the radiographer receiving a training in the hospital once every three months. The GP is also trained as a radiation protection expert and is responsible for radiation hygiene and safety together with the institute of Nuclear Services for Energy, Environment, and Health in the Netherlands.

### Questionnaire

Initially, information on the following variables was collected: sociodemographic variables (included age, sex, educational level, whether or not paid profession, health status, and status as an islander or tourist), previous x-ray experience, treatment experience after the x-ray examination (whether or not the patient received treatment as well as whether that treatment was received in general practice, by the GP or in hospital, by the specialist) and health status. The survey instrument was based on the Dutch version of the Patient Satisfaction Questionnaire III (PSQ.NL), a reliable instrument with adequate validity in hospital settings (e.g., oncology, surgery, cardiology departments) [26]. The PSQ.NL measures, in contrast to the original PSQ, satisfaction in general, as well as satisfaction with the technical quality of the GPs, interpersonal skills, and accessibility of care. However, our focus was on satisfaction with the technical and interpersonal skills of the GP as well as overall satisfaction with the service.

We selected one general satisfaction question, five medical technical questions and six interpersonal questions, beginning with PSQ questions were directly usable without adaptation, and then the ones that were usable with a slight modification. The general satisfaction question (1) was specifically adapted to address the x-ray service instead of the medical care. Two questions (8: I would rather go to the hospital for an x-ray and 12: Taking x-rays is a task for the hospital) replaced the original PSQ question (7: I think my doctor’s office has everything needed to provide complete care.; and we added the brand new question 2: I could choose whether to get an x-ray in the GP surgery or in hospital) (Additional file 1: Table S1). Our questionnaire is completed with four mirror questions (Additional file 1: Table S2).

The questions were answered using a 5-point Likert scale (i.e., [strongly] disagree, neutral, and agree [strongly]) and a no-opinion possibility. Answers in this last category were excluded from the statistical analyses.

To investigate possible biases in answering the questionnaire questions, we applied the matched pair method using four questions and their mirror questions. The question and mirror questions had opposite ends. Answers to negatively posed PSQ-questions were re-coded on a positive scale such that a high score corresponded to a positive attitude. A principal component analysis on the answers of the questions with a orthogonal rotation (varimax) with Kaiser normalization, revealed that the medical technical and interpersonal subscales were loaded in the right factors with Cronbach’s alfas of 0.76 each.

In addition, the benefits of the service were listed and patients were asked to rate each benefit in terms of its value to themselves (Additional file 1: Table S3).

### Statistical analysis

All analyses were performed with IBM SPSS Statistics for Windows, Version 20.0 (IBM Corp., Armonk, NY, USA). To determine the representativeness of the sample, age, sex, and island residency (yes or no) were compared with the research population, i.e., all patients that had an x-ray on the island during the research period, between June 1, 2007 and June 1, 2009. Data were presented as means of the sums of the sub-scale questions and standard deviations or as number (percentage). Analysis of variance (ANOVA) was used to analyze the differences between the means of the satisfaction subscales and the patient characteristics.

### Ethics statement

By asking patients for informed consent our study is conform the Medical Research Involving Human Subjects Act (WMO) in the Netherlands and does not need to be approved by a medical ethics committee.

## Results

### Respondent characteristics

Of the 587 patients invited to participate in the study, 381 returned the questionnaire (response proportion 65 %). The characteristics of the respondents are summarized in Table 1. The youngest patient was 5 years old and the oldest 101 years. The majority of patients (69 %) were island residents, with 31 % being non-islanders, these as well as gender and age were consistent with the ratios of all patients that had an x-ray on the island during the research period, between June 1, 2007 and June 1, 2009.

**Table 1:**
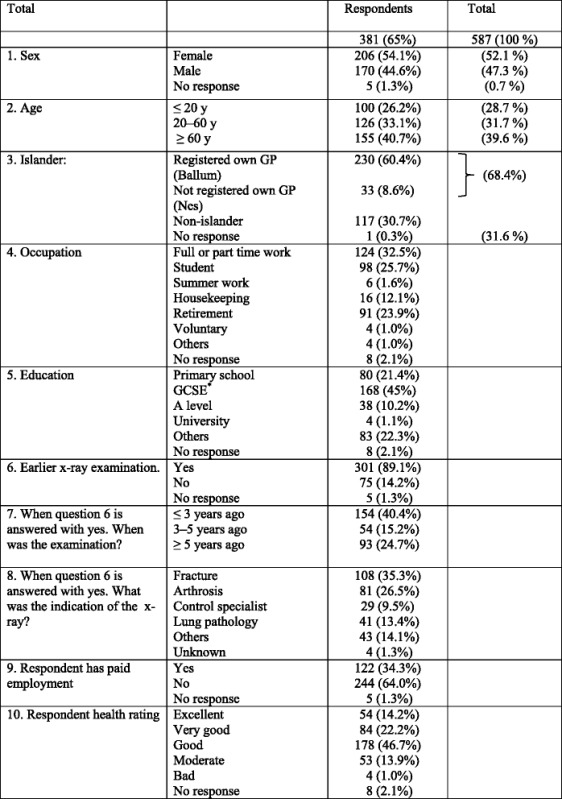
Characteristics of respondents

^*^ *GCSE* General Certificate of Secondary Education

Most patients were educated to at least secondary school level and were not in paid employment. The main indication for x-ray was suspected fracture (35.3 %). The majority (89.0 %) had previously had an x-ray examination.

### Patient satisfaction

The majority of patients (90.0 %) were very satisfied with the general practice teleradiology facility and with the extra medical care they received, scoring ≥ 4 on question 1 (Additional file 1: Table S4). The preferred arguments in favor of teleradiology were: “I liked the fact that I could stay on the island or at home” (83 %); “It took me no travel time” (68 %); and “This runs more quickly than in the hospital” (67 %) (Additional file 1: Table S5). Satisfaction with the technical skills of the doctor providing the teleradiology service was 22.4 ± 3.7 and satisfaction with the interpersonal manner of the doctor was 26.8 ± 3.8, out of maximum scores of 25 and 30, respectively (Table 2).

**Table 2:** Patient satisfaction

Questionnaire: Subscales	Subscales Mean ± SD	Number of observations	95 % Confidence interval	Number of items	Cronbach’s alpha
			Lower–Upper		
Satisfaction about interpersonal manner	26.8 ± 3.8	345	26.4–27.2	6	0.764
Satisfaction about technical quality	22.4 ± 3.7	355	22.0–22.7	5	0.761

Table 3 shows the satisfaction subscales by patient characteristics. ANOVA outcomes indicated a significant difference (*p* < 0.001) for islander patients and non-islander patients on both scales. Differences in satisfaction did not exist by sex, whether patients had previously had an x-ray, whether patients received treatment, whether that treatment was in general practice or hospital, level of education, health status, occupation, or between the two island general practices (*p* > 0.05 for all). However, satisfaction with interpersonal skills was significantly different for several variables (*p* < 0.001), with greater satisfaction for non-traumatic indications (versus traumatic indications), among islanders (versus non-islanders), and with advancing age (over 60 years > 20–60 years > less than 20 years) (Table 3).

**Table 3:** Patient satisfaction by sociodemographic variables

		Satisfaction with interpersonal manner			Satisfaction with technical quality		
		Mean ± SD	*n*	ANOVA	Mean ± SD	*n*	ANOVA
Total		26.4 ± 3.9	345		22.4 ± 3.7	355	
Sex	Female	26.6 ± 3.6	183	0.563	22.6 ± 3.4	189	0.247
	Male	26.9 ± 4.1	157		22.1 ± 4.1	161	
Age	≤20 y	24.6 ± 4.7	93	<0.001	21.5 ± 3.7	96	0.002
	20–60 y	27.1 ± 3.0	116		22.1 ± 4.2	121	
	≥60 y	28.1 ± 3.0	136		23.2 ± 3.3	138	
Islander	Yes	27.7 ± 3.1	237	<0.001	23.0 ± 3.2	244	<0.001
	[Registered own GP]	[27.7]		[0.845]	[23.0]		[0.982]
	[Not registered own GP]	[27.6]			[23.0]		
	No	24.9 ± 4.5	108		20.9 ± 4.3	111	
Employment	Paid	26.7 ± 3.0	118	0.972	22.3 ± 3.8	125	0.812
	Unpaid	26.7 ± 4.2	222		22.5 ± 3.8	225	
X-ray history	Yes	26.9 ± 3.6	264	0.162	22.4 ± 3.7	264	0.447
	No	26.2 ± 4.7	67		22.1 ± 3.5	67	
X-ray indication: Trauma	Yes	25.9 ± 4.2	177	<0.001	21.8 ± 3.9	182	0.004
	No	27.7 ± 3.1	168		22.9. ± 3.5	173	
Treatment	Yes	26.7 ± 3.8	215	0.837	22.2 ± 4.0	221	0.331
	No	27.0 ± 3.9	130		22.6 ± 3.2	134	
Treatment location	General Practice	26.8 ± 3.8	128	0.448	22.5 ± 3.6	130	0.141
	Hospital	26.3 ± 3.8	79		21.6 ± 4.5	83	
Health status	Good	27.7 ± 3.8	290	0.069	22.3 ± 3.7	304	0.715
	Moderate	26.6 ± 3.9	51		22.6 ± 3.8	55	
Education level	Low	27.9 ± 3.9	72	0.155	22.8 ± 3.3	74	0.461
	Medium	27.0 ± 3.0	154		22.5 ± 4.2	157	
	High	26.7 ± 3.0	33		22.5 ± 3.5	33	

## Discussion

This study shows that the majority of patients (90 %) having an x-ray taken in primary care on Ameland were very satisfied with the service overall and welcomed its introduction. Moreover, satisfaction with both the technical quality and the interpersonal manner were also very high, with neither sex, health status, level of education, previous in-hospital x-ray nor treatment by the GP influencing satisfaction. Island residents were more satisfied than non-residents with both the technical and interpersonal aspects of the service. Thus, the teleradiology service offered in primary care was well received on the Dutch island of Ameland. We believe that, because of the near complete isolation of the population of Ameland, the service facilitated continuity of the doctor-patient relationship for island residents. However, it was clear that the ability to stay on the island was the most important argument in favor of teleradiology, regardless of whether the patient was an islander or not.

Elderly patients and those with non-trauma indications were also more satisfied than younger patients and those with a trauma indication. Although this was true of both satisfaction subscales, the effect was most pronounced on the interpersonal scale for both age and indication. A possible explanation for the different satisfaction outcomes between trauma and non-trauma patients might be the different approach of the GP to the patient. For patients with trauma, the service tends to be more hurried and formal: to ensure accuracy and prevent complications, the GP focus is on providing quick instructions and checks followed by action and conclusion before terminating the consultation. This is a non-standard consultation technique in general practice. In contrast, a session with a non-trauma patient tends to begin with an introduction and then action, which is part of the normal GP-patient conversation that forms the basis of the doctor-patient relationship.

Our findings may be generalizable to other general practices since the current political strategy of the Western governments is to close hospitals, especially in the periphery. This implies that more and more patients will live in the future at a greater distance from the hospital and general practices becoming more remote. Our findings will be relevant for these remote general practices.

### Strengths and limitations

This is the largest study of patient satisfaction with teleradiology in primary care, adding additional information to our existing knowledge base in this area. In addition, it is the first study that investigates patient satisfaction in general and the technical quality and interpersonal manner during their experience of the teleradiology service. Another notable strength is the response proportion of 65 %.

Limitations of the study are the cross sectional design, it is a study at on specific point in time and we do not know the level of patient satisfaction before the introduction of the teleradiology service. Satisfaction with referral to the mainland hospital for an x-ray is also unknown. Both these facts preclude meaningful comparison. Therefore, we cannot conclude whether patient satisfaction improved after the introduction of the teleradiology service, or whether there was truly greater satisfaction than with the usual hospital service. Nevertheless, the most important perceived benefit with the teleradiology service in the general practice was the fact that patients could stay on the island. Future studies in this area might benefit from a before and after comparison to confirm our argument.

A further limitation is, the questionnaire is not completed directly after the x-ray is taken but after a short period which can – in theory - provide a bias. Also the fact that a validated questionnaire did not exist, so that we had to adapt the original PSQ-NL questionnaire is a limitation as well as the relatively high percentage of patients not answering certain items.

Another limitation is the concept of satisfaction. It is known that surveys of patients’ satisfaction often fail to distinguish between individual doctors because most of the variation in doctors is due to differences between patients and random error rather than differences between doctors. Measures related to patients’ experience discriminate more effectively between practices than do measures of general satisfaction [27]. However when we started our study the Consumer Quality (CQ) -questionnaire was not yet sufficiently developed [28].

### Comparison with existing literature

As mentioned in the background, we found just one article concerning teleradiology and patient satisfaction in general practice. However, that study did not distinguish between different subscales of satisfaction. The article concluded that patients were satisfied with the new facility and particularly appreciated the facility nearby [7]. This is consistent to our finding that, when using the teleradiology service, patients preferred the ability to remain on the island, the short travel time, and the faster service time in comparison with the hospital service.

In contrast to the limited research into patient satisfaction of teleradiology in primary care, more research has been performed into patient satisfaction of telemedicine in general [13–16, 20] and teledermatology in particular [17–19]. Nevertheless, each of these studies are deficient in some way, having small samples, low response rates, short investigation periods, or narrow definitions of satisfaction [18, 20]. Our study resolves these issues, providing a large sample with an acceptable response proportion over a long investigation period, while using clear definitions and measures of satisfaction.

It is well known from patient satisfaction studies of GPs that age, being seen by the same or usual doctor, education, sex, and health status can affect satisfaction with interpersonal communication [22–25, 29]. In our study, we additionally identified that satisfaction was different for technical quality and interpersonal manner. Indeed, satisfaction with the interpersonal manner was strongly affected by the patients’ age, whether they were an islander, and the indication for x-ray (trauma or not), while satisfaction with the technical quality was also strongly affected by whether they were an islander or not, but was less strongly influenced by age or indication. This distinction between island and non-island residents does not exist in the literature, which has only distinguished registered patients from others. Our data was also notable for the lack of influence of patient education status, health status, or sex on satisfaction levels, a fact that contrasts with the existing literature on satisfaction with GPs. This may however be an artefact of the specific period for which we carried out our study.

### Implications for research and practice

Future research should assess whether our finding that patients appreciate the teleradiology service also holds true for less remote general practices. In addition, more research is needed to assess the influence of the continuity of the doctor-patient relationship on patient satisfaction this together with the development of a patient satisfaction survey focused on the use of hospitals facilities in the general practice. Further research may also benefit from the inclusion of a Consumer Quality Index [28, 30–32] to advance our understanding of the patient experience.

## Conclusion

This study completes our research into the pilot teleradiology service in primary care on the Dutch island of Ameland. The introduction of teleradiology reduced the number of missed fractures and unnecessary referrals to the hospital and led to an increase in fracture treatment by the GP [12]. Moreover, it resulted in considerable cost reductions for patients (111 k euro per year) and health insurance companies (minimum 89 k euro per year) [11]. This study adds to these results, by showing that patients appreciated the teleradiology service. Thus, we conclude that the teleradiology is a suitable candidate secondary care facility for transfer to primary care, especially for patients who live at a considerable distance from hospital.

## Additional file

Additional file 1: Table S1. Patient satisfaction questions. **Table S2.** Mirror Questions. **Table S3.** Patients’ perceptions and expectations. **Table S4.** Results patient satisfaction questions. **Table S5.** Results: Patients’ perceptions and expectations. (DOCX 24 kb)

## Abbreviations

GP: General practitioner
PSQ: Patient Satisfaction Questionnaire
CQ: Consumer Quality
ANOVA: Analysis of variance
