# Supplementary material for: Patient satisfaction with a teleradiology service in general practice
Source: BMC Fam Pract. 2016 Feb 10;17:17. doi: 10.1186/s12875-016-0418-y (PMC4748486; doi:10.1186/s12875-016-0418-y)
Supplement: Additional file 1: Table S1. — Patient satisfaction questions. Table S2. Mirror Questions. Table S3. Patients’ perceptions and expectations. Table S4. Results patient satisfaction questions. Table S5. Results: Patients’ perceptions and expectations. (DOCX 24 kb) [file 12875_2016_418_MOESM1_ESM.docx]

Additional file 1

Table S1: Patient satisfaction questions

| Modified Questionnaire Item | Changes from Original Patient Satisfaction Questionnaire III (bracketed number indicates the original PSQ item) |
| --- | --- |
| General Satisfaction | |
| **1. I am very satisfied with the radiology instrument and the additional medical care I receive** | ***(****3). I am very satisfied with the medical care I receive* |
| **Interpersonal Satisfaction** | |
| **2.**.**I could choose whether to get an x-ray in the GP surgery or in Hospital** | (*New question)* |
| **3. The GP spends plenty of time with me** | *(38)* *Doctor is replaced by GP* |
| **4. The GP who treats me has a genuine interest in me as a person** | *(14)* *Doctor is replaced by GP* |
| **5. The GP listens carefully to what I have to say** | (*36)* *Doctor is replaced by GP* |
| **6. They did their best to keep me free from worrying** | (*39) Doctors always do their best to keep me free from worrying* |
| **7. All things considered, the medical care I receive is excellent** | (*35)* *All things considered, the medical care I receive is excellent* |
| **X-Ray Medical Technical Satisfaction** | |
| **8**. **I would rather go to the hospital for an x-ray** | *Newly developed from: (7) I think my doctor’s office has everything needed to provide complete care.* |
| **9. The GP makes me wonder if his or her diagnosis is correct** | (*10)* *Doctor is replaced by GP* |
| **10*.* I have some doubts about the ability of the GP who treats me** | (*37)Doctor is replaced by GP* |
| **11. There are some things about the medical care I received that could be better** | (*26*) *There are some things about the medical care I receive that could be better* |
| **12. Taking x-rays is a task for the hospital** | *Newly developed from: (7) I think my doctor’s office has everything needed to provide complete care.* |

Table S**2: Mirror Questions**

| **3. The GP spends plenty of time with me** | **13 . Those who provide me medical care hurry too much when he or she treats me** |
| --- | --- |
| **5 The GP listens carefully to what I have to say** | **14. There was no opportunity for me to ask questions** |
| **10 I have some doubts about the ability of the GP who treats me** | **15.The doctor who treats me is competent and well-trained in the x-ray examination** |
| **12. Taking x-rays is a task for the hospital** | **16.** **I am pleased with the x-ray facility and the extra treatment** |

Table S**3: Patients’ perceptions and expectations**

| **What was important to you when the x-ray was made in the general practice?** |
| --- |
| **I had the result immediately** |
| **I was shorter uncertain** |
| **I had shorter pain and discomfort** |
| **The treatment has been deployed faster** |
| **I saved travel costs** |
| **It took me no travel time** |
| **I lost less spare time** |
| **It took me no loss of time or revenues** |
| **I liked the fact that I could stay on the island or at home** |
| **This runs more quickly than in the hospital** |
| **I got the same quality of care as in the hospital** |
| **I saved costs** |

Table S4: Results patient satisfaction questions

|  | **strongly agree** | **agree** | **neutral** | **disagree** | **strongly disagree** | **no opinion** |
| --- | --- | --- | --- | --- | --- | --- |
| **General Satisfaction** | | | | | | |
| **1. I am very satisfied with the radiology instrument and the additional medical care I receive** | 80.8% | 9.2% | 3.4% | 1.8% | 1.3% | 3.4% |
| **Interpersonal Satisfaction** | | | | | | |
| **2.** **I could choose whether to get an x-ray in the GP surgery or in Hospital** | 54.3% | 6.0% | 16.8% | 3.1% | 13.9% | 5.8% |
| **3. The GP spends plenty of time with me** | 82.4% | 8,4% | 3.4% | 1.0% | 0.8% | 3.9% |
| **4. The GP who treats me has a genuine interest in me as a person** | 74.0% | 12.3% | 6.6% | 1.3% | 1.3% | 4.5% |
| **5. The GP listens carefully to what I have to say** | 78.0% | 11.8% | 3.4% | 0.8% | 1.0% | 5.0% |
| **6. They did their best to keep me free from worrying** | 75.3% | 13.9% | 5.5% | 0,5% | 0.8% | 3.9% |
| **7. All things considered, the medical care I receive is excellent** | 46.7% | 18.4% | 24.1% | 1.8% | 3.4% | 5.5% |
| **X-Ray Medical Technical Satisfaction** | | | | | | |
| 8**. I would rather go to the hospital for an x-ray** | 2.6% | 4.7% | 7.9% | 5.5% | 75.6% | 3.7% |
| **9. The GP makes me wonder if his or her diagnosis is correct** | 3.4% | 4.2% | 6.0% | 6.3% | 74.8% | 5.2% |
| **10. I have some doubts about the ability of the GP who treats me** | 2.6% | 0.8% | 5.5% | 6.0% | 80.6% | 4.5% |
| **11. There are some things about the medical care I received that could be better** | 5.2% | 7.6% | 10.0% | 10.0% | 63.5% | 3.7% |
| **12. Taking x-rays is a task for the hospital** | 1.8% | 6.0% | 11.5% | 9.7% | 66.1% | 4.7% |

Table S**5: Results: Patients’ perceptions and expectations**

| **What was important to you when the x-ray was made in the general practice?** | **Very important** | **Important** | **Neutral** | **Not important** | **Totally not important** | **No comments** |
| --- | --- | --- | --- | --- | --- | --- |
| **I had the result immediately** | 164(43%) | 88(23%) | 38(10%) | 4(1%) | 11(3%) | 76(20%) |
| **I was uncertain for less time** | 156(41%) | 84(22%) | 50(13%) | 4(1%) | 8 (2%) | 79(21%) |
| **I had shorter pain and discomfort** | 114(30%) | 76(20%) | 69(18%) | 8(2%) | 11(3%) | 103(27%) |
| **The treatment has been deployed faster** | 141(37%) | 84(22%) | 42(11%) | 11(3%) | 8 (2%) | 95(25%) |
| **I saved travel costs** | 149(39%) | 76(20%) | 49(13%) | 15(4%) | 46(12%) | 46(12%) |
| **It took me no travel time** | 183(48%) | 76(20%) | 34(9%) | 11(3%) | 23(6%) | 54(14%) |
| **I lost less spare time** | 130(34%) | 69(18%) | 49(13%) | 15(4%) | 46(12%) | 72(19%) |
| **I had no loss of time or revenues** | 95(25%) | 58(15%) | 72(19%) | 15(4%) | 46(12%) | 95(25%) |
| **I liked the fact that I could stay on the island or at home** | 244(64%) | 72(19%) | 11(3%) | 4(1%) | 8(2%) | 42(11%) |
| **This runs more quickly than in the hospital** | 168(44%) | 88(23%) | 31(8%) | 4(1%) | 11(3%) | 79(21%) |
| **I got the same quality of care as in the hospital** | 160(42%) | 88(23%) | 46(12%) | 4(1%) | 8(2%) | 76(20%) |
| **I saved costs** | 103(27%) | 69(18%) | 61(16%) | 15(4%) | 15(4%) | 118(31%) |
